# Enhanced Dewatering of Activated Sludge by Skeleton-Assisted Flocculation Process

**DOI:** 10.3390/ijerph19116540

**Published:** 2022-05-27

**Authors:** Jiahua Xia, Ting Rao, Juan Ji, Bijuan He, Ankang Liu, Yongjun Sun

**Affiliations:** 1 Nanjing Jiangbei New Area Public Utilities Holding Group Co., Ltd., Nanjing 210044, China; xiayu_610@sina.com (J.X.); raot@mail.ustc.edu.cn (T.R.); jj13814186775@163.com (J.J.); he13770509319@163.com (B.H.); 2 Nanjing Water Purification Environmental Research Institute Co., Ltd., Nanjing 211100, China; lakyx2@126.com; 3 College of Urban Construction, Nanjing Tech University, Nanjing 211800, China

**Keywords:** sludge conditioning, sludge dewatering, conditioner, fly ash, flocculation

## Abstract

Sludge dewatering is the fundamental process of sludge treatment. Environmentally friendly and efficient sludge conditioning methods are the premises of sludge to achieve dehydration reduction and resource utilization. In response to sewage plant sludge dehydration, fly ash (FA), polymerized aluminum chloride (PAC), and polymer sulfate (PFS) were studied separately to determine their sludge dehydration performance, and the effects of these three conditioner composite regulations on sludge dehydration properties were studied. Compared to the sludge treated only with conditioner, the average particle size of floc increased and the organic matter content in the filtrate decreased. The sludge dewatering efficiency after the conditioning effect is better than that after conditioning a single conditioner. After PFS conditioning with fly ash, the water content and specific resistance (SRF) of the sludge cake can be reduced to 76.39% and 6.63 × 10^10^ m/kg, respectively. The moisture content and specific resistance (SRF) of the sludge cake can be reduced to 76.10% and 6.91 × 10^10^ m/kg, respectively. The pH of the sludge and filtrate changed slightly after PAC conditioning with fly ash coupling. These results indicate that fly-ash coupled with PAC and fly-ash coupled with PFS are expected to become a novel and effective environmental protection combined conditioning method for sludge dewatering.

## 1. Introduction

Great things were achieved in the sewage treatment industry with the completion and operation of a large number of municipal sewage treatment plants [1]. The proliferation of wastewater treatment has led to the production of a large amount of activated sludge, which has gradually increased the load on sewage treatment plants, and wastewater treatment has become a key challenge for global water management [2]. However, the wastewater treatment industry has long been “heavy water and light sludge”, resulting in a serious lag of the water treatment industry in terms of sludge treatment level, technology and capital investment [3]. At present, the sludge generated by a large number of wastewater treatments cannot be effectively treated and disposed of, and the persistent heavy metals and persistent organic pollutants in the sludge will pose a great threat and challenge to the natural environment, such as atmosphere, water, and soil [4]. Among other things, the high moisture content of sludge becomes the bottleneck in sludge treatment and disposal, and the high moisture content and low bulk density of sludge pose problems in storage, transportation, and economic costs [5]. Therefore, the sludge must be dewatered immediately. Mechanical dewatering is widely used in wastewater treatment plants, but the efficiency of mechanical dewatering of sludge is limited due to the hydrophilicity of extracellular polymers [6]. Therefore, before mechanical dewatering, the sludge needs to be pre-treated to reduce the moisture content of the sludge and improve the sludge dehydration efficiency.

The commonly used sludge conditioning methods include physical conditioning, chemical conditioning, and biological conditioning. At present, physical conditioning is a sludge conditioning method that destroys the microbial cells in the sludge by physical methods, changes the structure of the sludge, and reduces the binding effect of the sludge and water, thereby releasing the internal water [7]. Although physical conditioning can reduce the moisture content of the sludge, each has its own advantages and disadvantages due to its different mode of action. Among them, thermal conditioning has obvious effects on improving the dewatering rate of most of the sludges, but the treatment effect on low-organic sludges is small [8]. Although the microwave treatment time is short, the heating temperature is low, the thermal efficiency is high, and the equipment is simple, the microwave treatment has requirements on the amount of sludge entering the sludge. Freeze conditioning can be carried out spontaneously, without chemical additives and inexpensively, but is limited in its scope. Ultrasonic conditioning has high efficiency and environmental protection and application prospects, but its working principle and complex effects need to be further discussed [9].

Overall, the high energy consumption and the high equipment costs of the physical conditioning restrict its large-scale use. Microbial conditioning promotes mutual coagulation and precipitation of colloidal suspensions in water and improves sludge dewatering performance. Microbial conditioning has powerful benefits such as: high efficiency, non-toxicity, no secondary pollution, biodegradation, compact sludge flakes and a wide range of applications [10]. However, the current research on such flocculants is not comprehensive, the research level is low, the mechanism of flocculants is not fully understood, and problems such as large dosage, high production cost, and poor suitability arise.

Chemical conditioning is widely used as the optimal choice among broad comparisons. In general, chemical conditioning is mainly divided into inorganic and organic polymer conditioning [11]. At present, the widely used inorganic chemical conditioning agents mainly include polyferric sulfate (PFS), polyaluminum chloride (PAC), polyaluminum ferric chloride (PAFC), and polyaluminum silicate (PASiC). Organic conditioners have different electrical properties. Cationic and amphoteric are more commonly used as flakes are often negatively charged [12]. The use of inorganic conditioning agents for chemical conditioning has the advantages of easy availability of raw materials, easy manufacture and low cost, and it is very effective in removing fine suspended particles in sludge [13]. These advantages greatly increase the amount of sludge, reduce the fertilization efficiency and calorific value of the sludge, and also bring heavy metals into the sludge, posing a threat to the environment. Organic polymer conditioners have many types, each with its own advantages [14]. However, the organic flocculants have the disadvantage that they are difficult to degrade and the residual monomers are toxic. There are certain disadvantages due to physical, chemical, and microbial conditioning alone, and combined conditioning technology has become a research focus in recent years [15]. Combined conditioning can reduce the compressibility, the amount of chemicals added, and the cost of conditioning, and improve the dewatering performance of the sludge [16]. The research and development of efficient, low-consumption, safe and stable flocculants is the direction and goal of flocculant research in view of the currently increasing water pollution and ever stricter environmental regulations.

This study was used for sludge pretreatment with PAC, PFS, and fly ash. Sludge ratio resistance SRF was used as an indicator of sludge dewatering properties with sludge. Filtrate pH, sludge pH, filtrate COD, sludge SEM, filtrate UV spectrum, and floc particle size were the most intuitive evaluation indices of dewatering performance. By examining the influence of different chemical conditioning agents on different indicators, the main influencing factors of the three aforementioned conditioners are optimized, the sludge conditioning mechanism of different conditioners is discussed, and the sewage treatment plant is measured in different ways. The effect of sludge dewatering performance is enhanced to provide a reference for sludge pretreatment technology of sewage treatment plants.

## 2. Materials and Methods

### 2.1. Materials

Polymeric ferric sulfate, ammonium molybdate, silver sulfate, ferric nitrate, cerium nitrate, cobalt nitrate, potassium sodium tartrate, and ascorbic acid were all of analytical grade and were purchased from Sinopharm Chemical Reagent Co., Ltd. (Shanghai, China). Mercury sulphate and potassium antimony tartrate were all of analytical grade and were purchased from Shanghai McLean Biochemical Co., Ltd. (Shanghai, China). The fly ash was taken from a coal chemical enterprise in Jiangsu. Cationic polyacrylamide (CPAM) was purchased from Jiangyin Huadong Water Treatment Co., Ltd. (Wuxi, China). The actual sludge water sample was taken from a sewage treatment plant in Jiangsu, and the original sludge quality is shown in Table 1.

### 2.2. Sludge Conditioning and Dehydration Experiments

Sludge conditioning experiments were carried out with fly ash, PAC, and PFS. For different coagulants, six groups of samples of 500 mL each were taken each time, and different concentrations of coagulants were added into them. DS is the abbreviation for Dry Solid Content. According to the pre-experiment, different fly ash dosages (e.g., 5, 10,15, 20, 25, 30 g/g DS) were selected. Different dosages of PAC (e.g., 50, 100, 150, 200, 250, 300 mg/g DS) and PFS (e.g., 50, 100, 150, 200, 250, 300 mg/g DS) were used to improve the sludge dewatering performance. After addition of the agent, we spun at 200 rpm for 30 s, then spun at 60 rpm for 3 min and let stand for 30 min before dehydration. After selecting the optimal fly ash dosage, the above steps were repeated for fly ash combined with PAC and fly ash combined with PFS.

Sludge dewatering adopts vacuum filtration. First, the wet filter paper is spread out in the cloth funnel. After removing the air bubbles, the dosed sludge was injected into the cloth hopper and the vacuum filtration pressure is maintained at 0.05 MPa. It was filtered for 2 min and the duration of the suction and the filtrate volume suctioned off during the corresponding time were recorded. After dosing and stirring, the pH of the sludge was measured. After the sludge was dewatered, the filtrate was taken out, and the pH of the filtrate and the COD of the filtrate, and the UV spectrum of the filtrate were measured. The SRF is calculated from the measurements taken during the dewatering of the sludge. After drying the sludge cake, moisture content, SEM, fractal dimension, and micrograph of the filter cake were measured.

### 2.3. Sludge Index Analysis Method

COD was measured with a Hach analyzer (COD analyzer, DR1010, Hach Company, Loveland, CO, USA); the particle size analysis of the sludge cake was determined with a laser particle size analyzer (laser particle size analyzer, Winner2018, Jinan Micro-Nano Particle Instrument Co., Ltd., Jinan, China); and the UV of the filtrate was measured with a UV/visible spectrophotometer (UV/visible spectrophotometer, UV-5500PC, Shanghai Yuanyan Instrument Co., Ltd., Shanghai, China) in the entire UV band (GB 6920-86).

## 3. Results

### 3.1. Effect of PFS Dosage on Sludge Dewatering Performance

**Figure 1:**
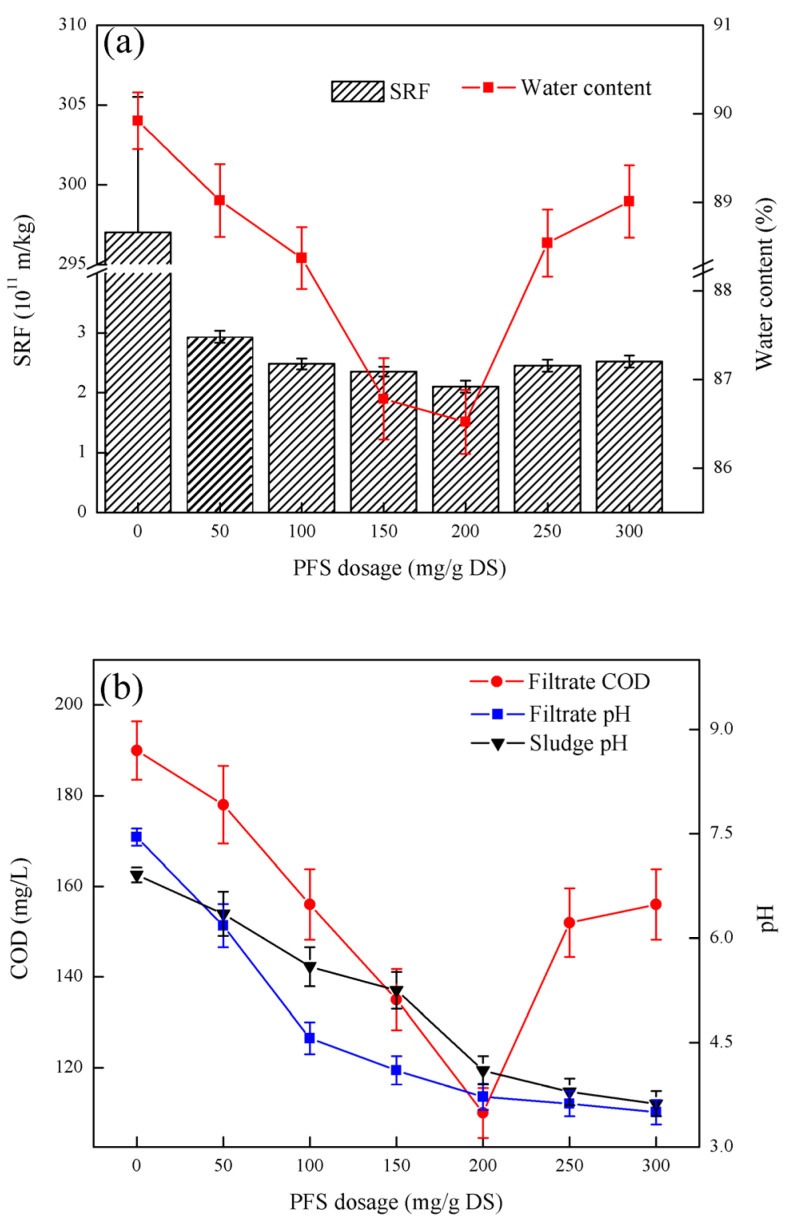
Effect of PFS dosage on sludge dewatering performance: (**a**) PFS dosage on SRF and filter cake moisture content, (**b**) PFS dosage on filtrate COD, filtrate pH and sludge pH.

As shown in Figure 1, the SRF of the sludge first decreased and then increased with increasing PFS dosage. When the PFS dosage is 200 mg/g DS, the lowest sludge specific resistance SRF is 2.10 × 10^11^ m/kg. The change trend of the moisture content of the filter cake is the same as that of the sludge specific resistance SRF. At the PFS dosage of 200 mg/g DS, the minimum moisture content of the filter cake was 86.52%. With increasing PFS dosage, the COD of the filtrate first decreased and then increased. When the PFS dosage is 200 mg/g DS, the minimum COD content of the filtrate is 110 mg/L. The pH of the filtrate decreased rapidly with the increasing in PFS dosage and then stabilized. When the PFS dosage was 300 mg/g DS, the pH of the filtrate was at least 3.50. The pH of the sludge decreased gradually with the increasing in PFS dosage. When the dosage of the PFS was 300 mg/g DS, the lowest sludge pH was 3.62.

Figure 1 shows that PFS can improve the filtration performance of the sludge. However, when the PFS dosage reached the optimum, the sludge specific resistance SRF did not improve significantly. When the PFS dosage exceeds 200 mg/g DS, the sludge resistivity SRF obviously increases, showing the inhibitory effect of sludge dewatering performance [17]. The reason is that when the PFS concentration is too high, the long-chain molecules may adhere to each other and cannot be stretched effectively, thereby reducing the total amount of adsorbed sludge colloidal particles and the flocculation efficiency. The tendency of the moisture content of the sludge cake to change was similar to the sludge specific resistivity SRF, which further indicated that excess PFS had an inhibitory effect on the sludge dewatering performance [18]. The COD of the filtrate first decreased and then increased because Fe^3+^ dissolved in water and neutralized the negative charge on the sludge surface, hence causing the sludge flocs to aggregate into large particles to settle and form hydroxide precipitates. The organic matter in the sludge remains in the sludge cake. When the PFS dosage is too high, the long chains of PFS will combine with each other, and the organic matter cannot be efficiently flocculated into large particles and becomes trapped in the sludge cake. The pH of the sludge and filtrate decreased because PFS was essentially an intermediate product of polynuclear hydroxyl complexes, and the mechanism of action was close to that of organic polymers. The adsorption of PFS and particulate matter is actually a surface complex coordination, and the surface hydroxyl groups will supplement unsaturated sites [19]. After adsorbing the particles on the surface of PFS, they will still absorb hydroxyl groups from the solution and continue the hydrolysis and precipitation process until they become saturated and become a hydroxide precipitation gel. Thus, the pH of the sludge and filtrate are acidic. After considering the three indicators of SRF, moisture content of the sludge cake, and COD in the filtrate, the optimal PFS dosage is 200 mg/g DS.

### 3.2. Effect of PAC Dosage on Sludge Dewatering Performance

**Figure 2:**
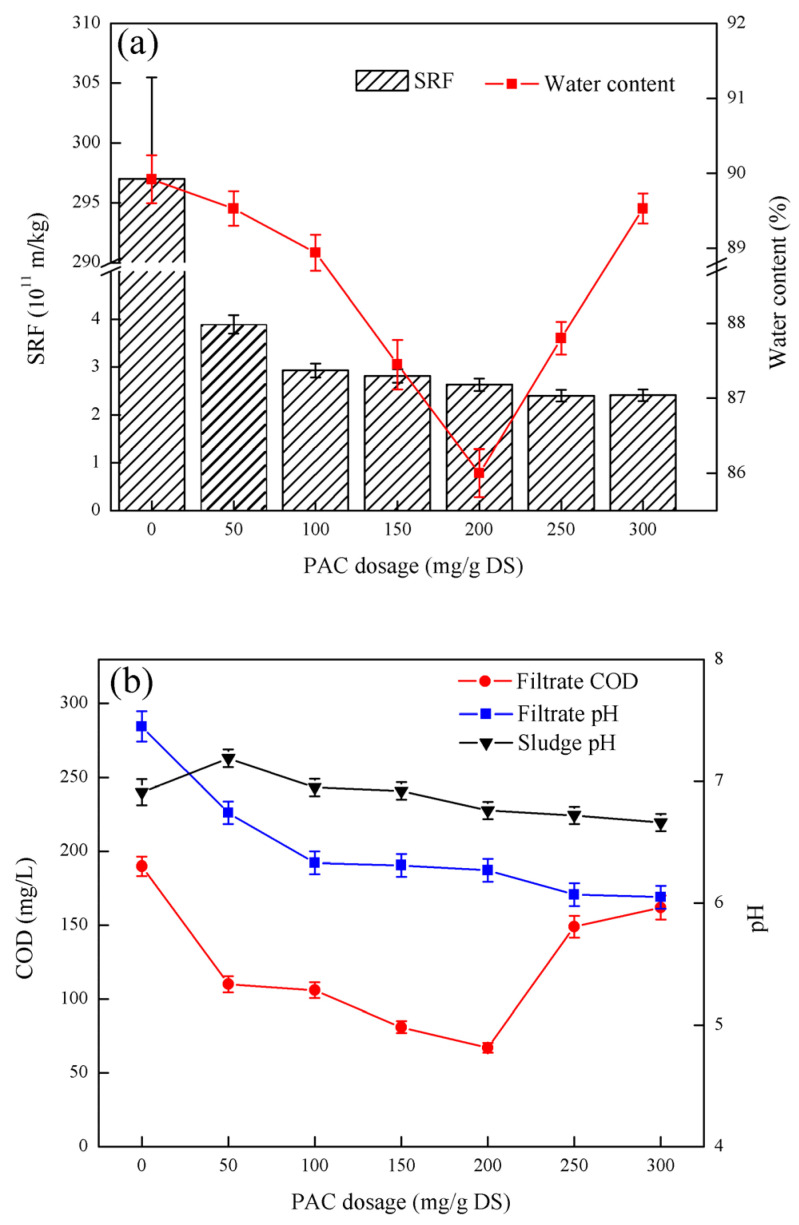
Effect of PAC dosage on sludge dewatering performance: (**a**) the effect of PAC dosage on SRF and filter cake moisture content, (**b**) the effect of PAC dosage on filtrate COD, filtrate pH and sludge pH.

As shown in Figure 2, with increasing the PAC dosage, the SRF of the sludge first decreased and then increased. When the PAC dosage was 200 mg/g DS, the lowest SRF of sludge specific resistance was 2.40 × 10^11^ m/kg. The change trend of the moisture content of the filter cake is the same as that of the sludge specific resistance SRF. When the PAC dosage was 200 mg/g DS, the minimum moisture content of the filter cake was 86.00%. With the increase in PAC dosage, the COD of the filtrate first decreased and then increased. When the PAC dosage was 200 mg/g DS, the minimum COD content of the filtrate was 67 mg/L. The pH of the filtrate decreased slowly with the increase in PAC dosage. When the PAC dosage was 300 mg/g DS, the pH of the filtrate was at least 6.05. The pH of the sludge decreased gradually with the increase in PAC dosage. When the PFS dosage was 300 mg/g DS, the lowest sludge pH was 6.66.

As shown in Figure 2, the addition of PAC can greatly reduce the specific resistivity SRF of the sludge, so that the sludge has good dewatering performance. When the sludge specific resistance SRF was reduced to 2.40 × 10^11^ m/kg, and the PAC dosage further increased, the sludge dewatering performance did not improve significantly, and the sludge specific resistivity had a gradually increasing tendency. Similar to the addition of excess PFS, excess PAC also exhibited an inhibitory effect on sludge dewatering performance because when the dosage is too high, the surface of the colloid may be covered by the inorganic PAC macromolecule [20]. When the two colloidal particles are close to each other, they cannot aggregate due to the mutual repulsion between the PAC macromolecules, thereby resulting in a “colloidal protective” effect. The flocculation effect is reduced or even restabilized. Therefore, part of the organic matter in the sludge cannot be retained in the sludge cake, hence increasing the COD of the filtrate when the amount of PAC is too high. The change trend of the moisture content of the filter cake is similar to that of the SRF, which further indicates that an adequate amount of PAC can promote the dewatering performance of the sludge [21]. The hydrolysis intermediate of PAC has a higher positive charge, and due to the stronger electrical neutralization and pollutants, PAC has less effect on the filtrate pH and sludge pH than PFS. Compared with PFS, the specific resistance of sludge conditioned by PAC was slightly higher than that of 2.10 × 10^11^ m/kg after conditioning by PFS because cell lysis helps to release intracellular water under acidic conditions, thereby further improving the sludge dewatering performance. After considering the three indicators of SRF, sludge cake moisture content and filtrate COD, the optimal PAC dosage was 200 mg/g DS.

### 3.3. Effect of Fly Ash Dosage on Sludge Dewatering Performance

**Figure 3:**
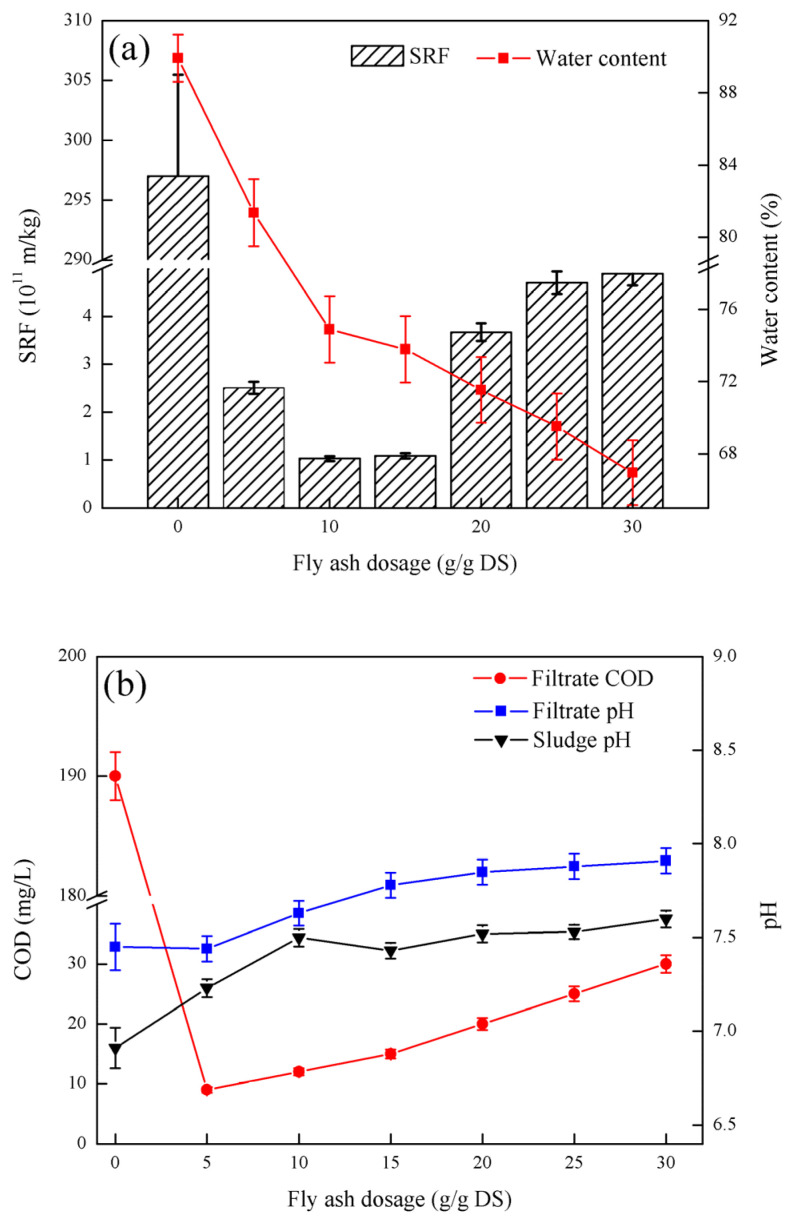
Effect of fly ash dosage on sludge dewatering performance: (**a**) the effect of fly ash dosage on SRF and filter cake moisture content, (**b**) fly ash dosage on filtrate COD and filtrate pH and sludge pH.

As shown in Figure 3, as the fly ash dosage increased, the SRF of the sludge first decreased and then increased. When the fly ash dosage is 10 g/g DS, the minimum SRF is 1.03 × 10^11^ m/kg. The moisture content of the filter cake gradually decreased with increasing fly ash dosing. When the fly ash dosage is 30 g/g DS, the minimum moisture content of the filter cake is 66.95%. The COD of the filtrate increased gradually with the addition of fly ash. When the fly ash dosage was 5 g/g DS, the COD content of the filtrate was at least 9 mg/L. The pH of the filtrate gradually increased with the fly ash dosing. When the fly ash dosage was 30 g/g DS, the pH of the filtrate was at its highest at 7.91. With increases in fly ash dosage, the pH of the sludge slowly increased. When the fly ash dosage was 30 g/g DS, the sludge pH was up to 7.60.

Figure 3 shows that fly ash can improve the dewatering performance of sludge. The raw sludge flocs are loose and few internal filtration channels are observed, and separating the water from the sludge is difficult, thereby making the filtration speed very slow. Forming a porous structure within the fly ash particles is easy. Due to its small particle size and strong surface adsorption capacity, a charge neutralization effect occurs after mixing with the sludge. When the surface tension is larger than the adsorption force of the sludge on the adsorbed water, the sludge adsorbed water can be separated. In addition, after mixing the fly ash with the sludge, it can absorb the sludge flocs and form a skeleton structure with its particles as the center, which reduces the compressibility of the sludge, builds a water filter channel for the sludge cake, and reduces the sludge viscosity, which significantly reduces the sludge dewatering time [22]. If the amount of fly ash is too large, the fly ash will accumulate itself, clogging the water filter channel and increasing the sludge resistivity. The trend in moisture content of fly ash and PAC and PFS conditioned sludge cakes is different, showing that the moisture content of conditioned sludge cakes continues to decrease. The reason is that the fly ash dosage is significantly increased compared to PAC and PFS, which significantly increases the total solid content in the sludge [23]. When the amount of fly ash reaches a certain level, the decrease in the moisture content of the sludge is caused only by the increase in the solid content and has nothing to do with the properties of the fly ash itself. Therefore, under high dosage, the moisture content of the filter cake shows a rapidly declining trend. The COD of the filtrate continues to increase because fly ash is a tiny ash particle discharged during the combustion of fuel, which contains unburned carbon, calcium silicate, and anorthite [24]. When the fly ash is added to the sludge, hydrolysis takes place, increasing the COD of the filtrate and making the solution weakly alkaline. Therefore, the pH of the sludge and filtrate slowly increases. With full consideration of economic benefits and dehydration performance, the fly ash was tested at 10g/g TS by coupling PFS and PAC.

### 3.4. Effect of Fly Ash Coupled with PFS on Sludge Dewatering Performance

**Figure 4:**
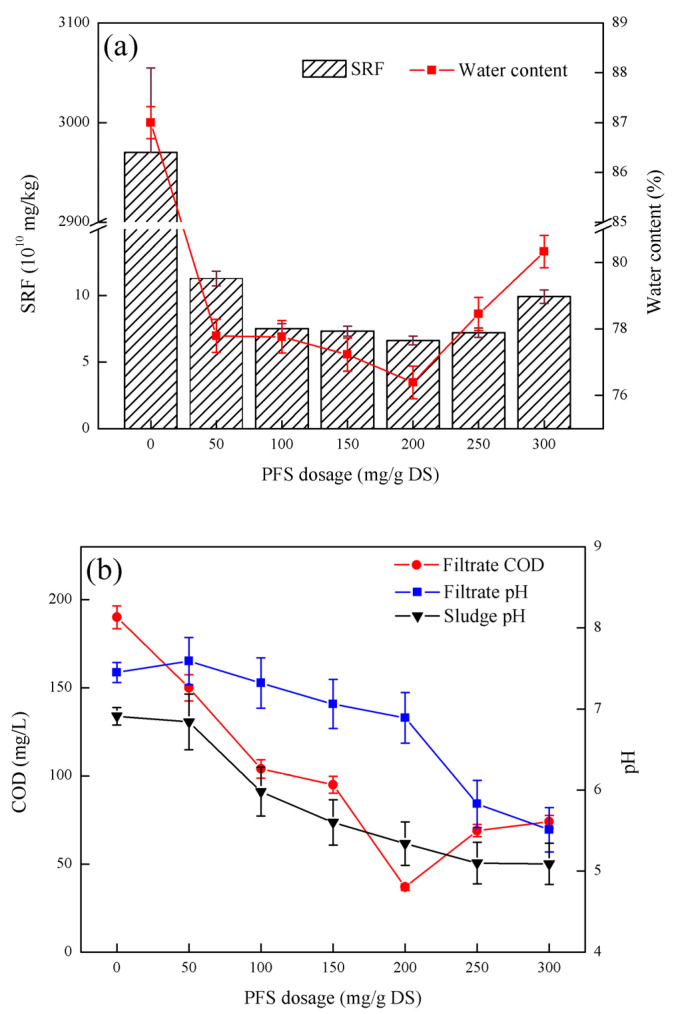
Effect of fly ash coupled with PFS on sludge dewatering performance: (**a**) the effect of PFS dosage on SRF and filter cake moisture content, (**b**) the effect of PFS dosage on filtrate COD, filtrate pH, and sludge pH Influence.

As shown in Figure 4, the sludge SRF first decreased and then increased with increasing PFS dosage. When the PFS dosage was 200 mg/g DS, the lowest specific resistance of sludge was 6.63 × 10^10^ m/kg. The moisture content of the filter cake first decreased and then increased again with increasing in PFS dosage. When the PFS dosage was 200 mg/g DS, the minimum moisture content of the filter cake was 76.39%. The COD of the filtrate first decreased and then increased with the PFS dosage. When the PFS dosage was 200 mg/g DS, the minimum COD content of the filtrate was 37 mg/L. The pH of the filtrate gradually decreased with increasing PFS dosage. When the PFS dosage was 300 mg/g DS, the pH of the filtrate was at least 5.51. With the increase in PFS dosage, the pH of the sludge gradually decreased and tended to be flat.

Figure 4 shows that fly ash coupled with PFS can significantly improve sludge filtration performance. Under the same conditions, fly ash coupled with PFS can improve the sludge dewatering effect. Fly ash provides an unobstructed water filtration channel and allows the PFS to play a better role by changing the microstructure of the sludge and its own adsorption performance [25]. When the PFS dosage is too high, the long-chain structure of the PFS itself sticks together, so that the electrostatic charge of the particles is positive, which leads to the stability of the particles, the specific resistance of the sludge, the moisture content of the filter cake, and the COD of the filtrate [26]. The changing trends of the filter cake pH and sludge pH were consistent with the addition of PFS alone, but the pH of sludge and pH of filtrate after PFS conditioning in combination with fly ash were greater than those after PFS conditioning alone. This phenomenon shows that PFS adsorbs hydroxyl groups from the solution to make the sludge acidic, and the calcium silicate and anorthite in the fly ash can neutralize a certain acidity after dissolving in water, which will affect the sludge pH and pH of the sludge can increase filtrate [27]. After considering the three indicators of SRF, sludge cake moisture content, and filtrate COD, the optimal PFS dosage became 200 mg/g DS.

### 3.5. Effect of FA-Coupled PAC on Sludge Dewatering Performance

**Figure 5:**
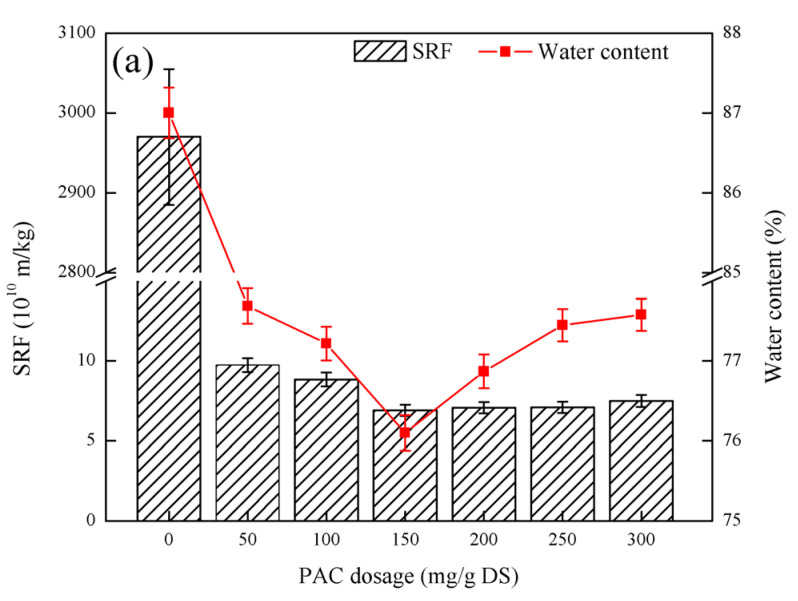

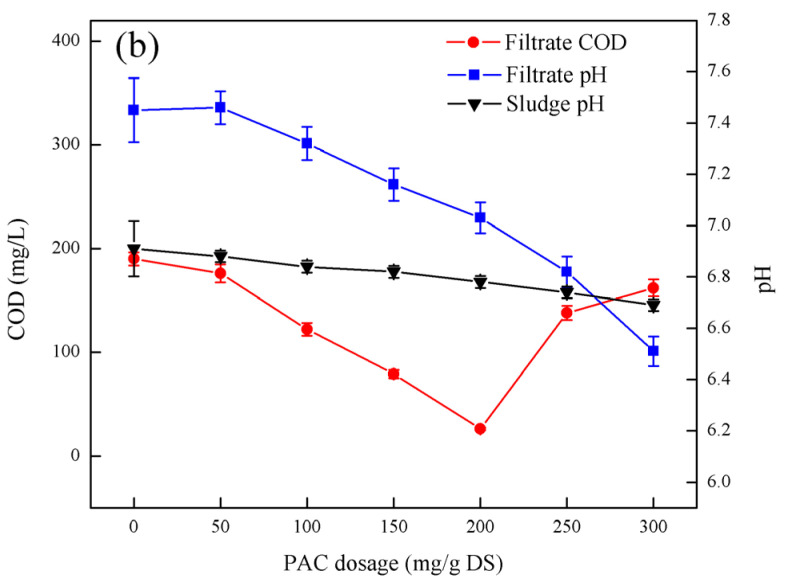
Effect of fly ash coupled with PAC on sludge dewatering performance: (**a**) the effect of PAC dosage on SRF and filter cake moisture content, (**b**) the effect of PAC dosage on filtrate COD, filtrate pH, and sludge pH Influence.

As shown in Figure 5, with the increase in PAC dosage, the sludge SRF first decreased and then increased. When the PAC dosage was 150 mg/g DS, the lowest SRF value was 6.91 × 10^10^ m/kg. With increasing PAC dosage, the moisture content of the filter cake first decreased and then increased again. When the PAC dosage was 150 mg/g DS, the minimum moisture content of the filter cake was 76.10%. The COD of the filtrate first decreased and then increased with the increase in PFS dosage. When the PAC dosage was 200 mg/g DS, the minimum COD of the filtrate was 26 mg/L. The pH of the filtrate decreased gradually with the increase in the PAC dosage and tended to be gentle. When the PAC dosage was 300 mg/g DS, the pH of the filtrate was at least 7.31. The pH of the sludge decreased gradually with the increase in PAC dosage. When the PAC dosage was 300 mg/g DS, the pH of the sludge was the lowest at 7.04.

Figure 5 shows that the fly ash coupled with PAC can improve the filtration performance of the sludge. In the process of sludge conditioning by fly ash coupled with PAC, the hydrolyzate interacts with the colloid in the sludge through charge neutralization and adsorption bridging, which reduces the surface charge of the sludge particles, aggregates the sludge particles into clusters, and increases the concentration of sludge particles. Larger sludge particle size increases sludge density. By forming a “skeleton”, the fly ash reconstructs the sludge structure, plays a supporting role in the subsequent dehydration process, and provides a water filter channel [28]. When the PAC dosage is too high, the long-chain structure of the PAC itself sticks to each other, which makes the electrostatic charge of the particles positive, which leads to the stability of the particles, blocks the fine pores formed by fly ash, and increases the specific resistance of the sludge [29]. The trend of change in the moisture content of the sludge cake was similar to that of the sludge resistivity SRF, further indicating that excess PFS had an inhibitory effect on sludge dewatering performance. With a suitable PAC dosage, the organic matter can be retained effectively and the COD of the filtrate gradually decreases [30]. If the PAC dosage is too high, the organic matter in the sludge cannot be retained effectively. In addition, the fly ash contains unburned carbon. When the fly ash is added to the sludge, hydrolysis takes place, increasing the COD of the filtrate. The change trend of filter cake pH and sludge pH is consistent with the trend of adding PAC alone and coupled with PFS by fly ash, but the pH of the sludge and filtrate after the conditioning of fly ash coupled with PAC is greater than that of adding PFS alone and coupled with fly ash. The sludge pH and filtrate pH after PFS conditioning indicate that the hydrolysis intermediate of PAC has a higher positive charge than PFS, which is mainly due to the strong electric neutralization and contaminant interaction [31]. Calcium silicate and anorthite in fly ash can neutralize certain acidity after being dissolved in water and can also increase the pH of sludge and filtrate. After considering the three indicators of SRF, sludge cake moisture content, and filtrate COD, the optimal PAC dosage was 200 mg/g DS.

### 3.6. Floc Structure after Sludge Conditioning

**Figure 6:**
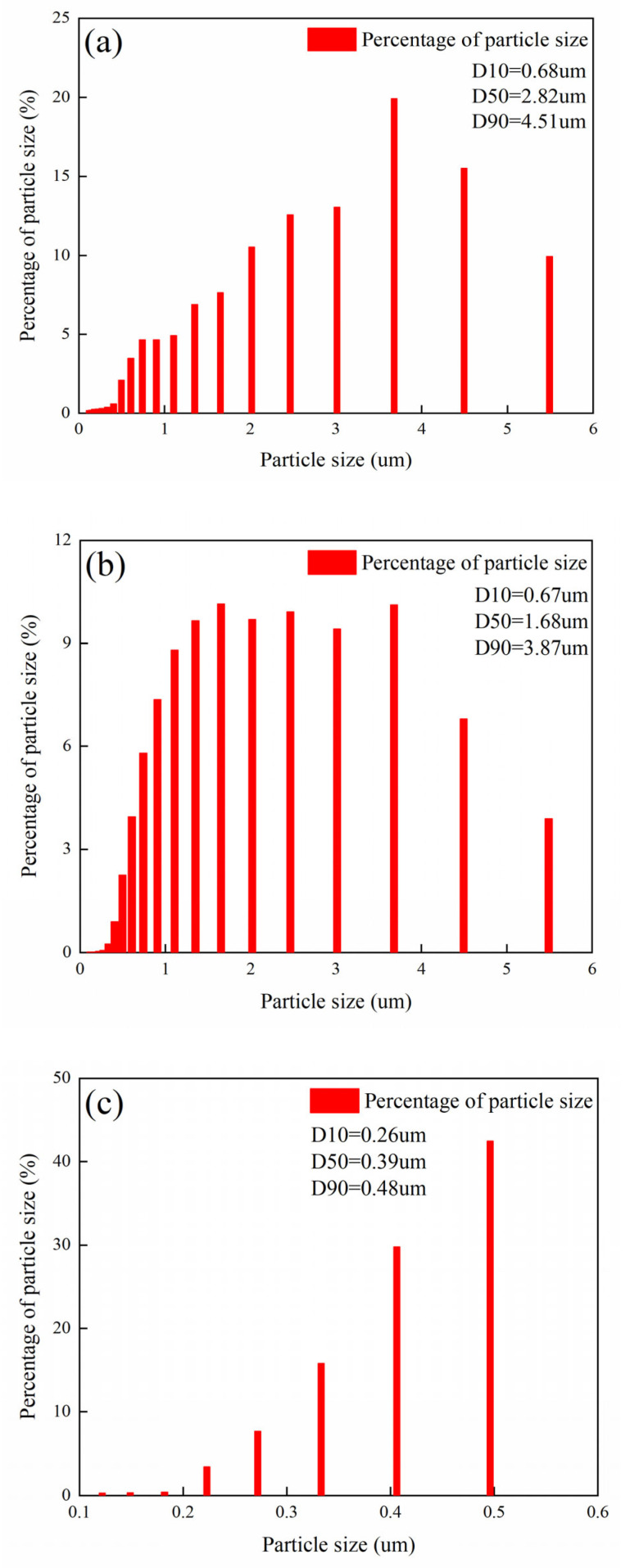

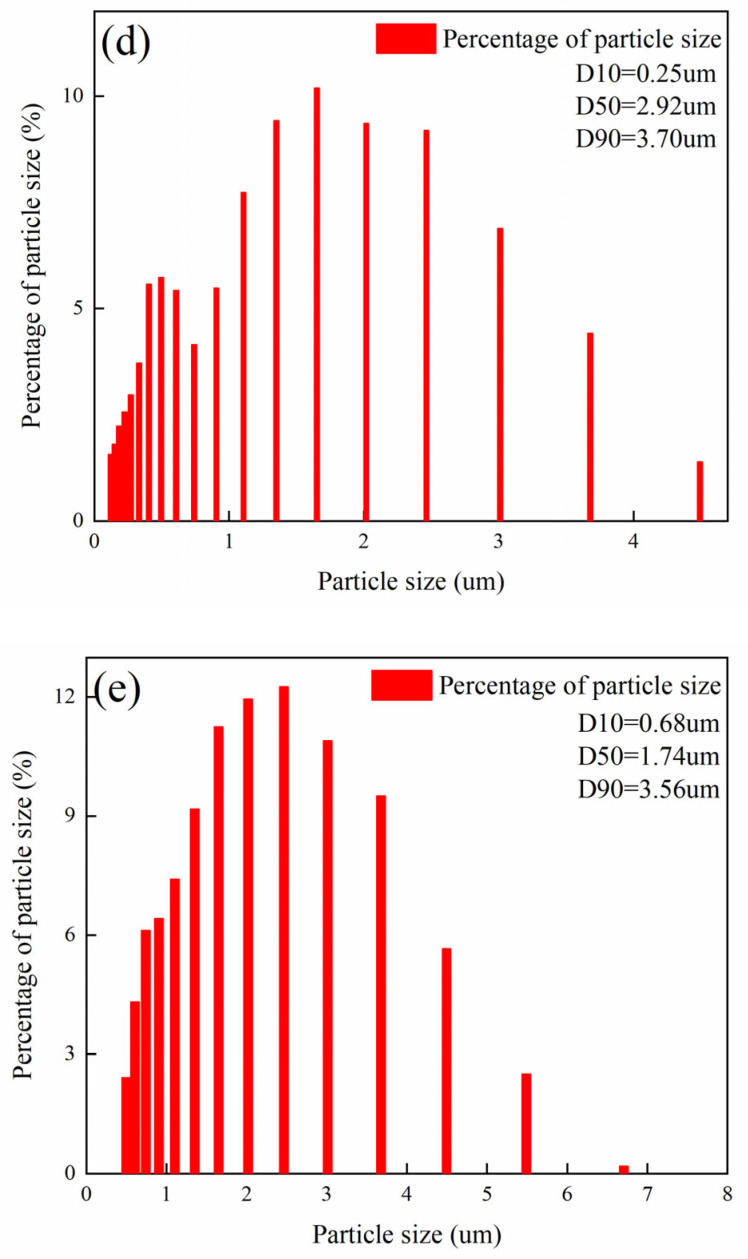
Floc size distribution of sludge conditioned with various flocculants under the optimal dosage conditions: (**a**) PFS, (**b**) PAC, (**c**) fly ash, (**d**) fly ash + PFS, (**e**) Fly Ash + PAC.

As shown in Figure 6, after adding different conditioners, the particle size distribution follows the same rule: with the increase in particle size, it all shows a trend of first increasing and then decreasing. The D50 of the sludge conditioned by PFS, PAC, FA, fly ash coupled with PFS, and fly ash coupled with PAC were 2.82, 1.68, 0.39, 2.92, and 1.74 µm, respectively. The median particle size of the sludge conditioned by fly ash coupled with PFS was significantly smaller than other conditioners.

Figure 6 shows that the flocs of the raw sludge are loose. The addition of the compound conditioner can neutralize the negative charge on the surface of the flocs, reduce the repulsive force between colloidal ions, and make the flocs prone to collision and aggregation, thus forming flocculation particles with large particle size and dense structure. By comparing the size of the sludge flocs under the optimal dosage of the five conditioners, the size relationship of the median particle size of the sludge particles after conditioning by the five conditioners is: fly ash coupled with PFS > fly ash Coupling PAC > PFA > PFS > PAC. The size of the flocs conditioned by the PFS conditioner is larger than that of the PAC conditioner. During the coagulation process, PFS tends to hydrolyze to form insoluble hydroxide precipitates because the hydrolysis rate of PFS is higher than that of PAC [32]. The order of the median particle size of flocs does not completely correspond to the sludge conditioning performance, indicating that the particle size formed during the sludge conditioning process is not the only factor that affects the sludge dewatering performance [33]. The long-chain structure of PFS and PAC helps to form larger floc size.

### 3.7. Effects of Different Conditioners on UV of Filtrate

**Figure 7:**
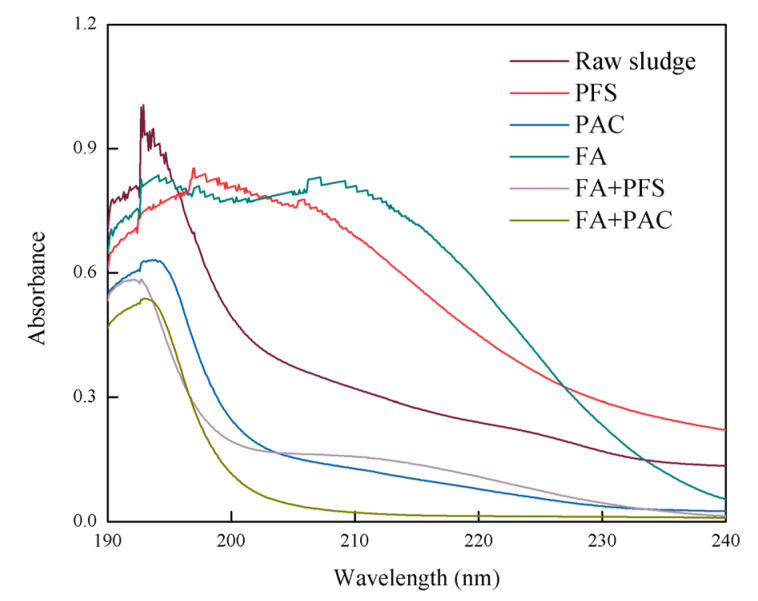
Effects of different conditioners on UV of filtrate.

As shown in Figure 7, the raw sludge and the conditioned sludge filtrate show clear absorption peaks between 190 nm and 230 nm. The peak of the original sludge is 192.9 nm, and the absorbance is 4.0223. The peak of the sludge filtrate after PFS conditioning was 197.9 nm, and the absorbance was 3.3556. The peak of the sludge filtrate after PAC conditioning was 193.6 nm, and the absorbance was 2.5271. The peak of the sludge filtrate conditioned by fly ash is 194.1, and the absorbance is 3.342. The peak of the sludge filtrate conditioned by fly ash coupled with PFS is 192.7 nm, and the absorbance is 2.3378. The peak of the sludge filtrate conditioned by fly ash coupled with PAC was at 193 nm, and the absorbance was 2.1510. The absorbance of the conditioned sludge was less than 4.0223. Among them, the absorbance of sludge filtrate conditioned by fly ash coupled with PAC was lowest.

Figure 7 shows that the raw sludge has clear UV absorption peaks at 190–230 nm, of which 190–230 nm is the characteristic band of organic acids and aromatic compounds. Therefore, many refractory aromatic compounds were observed in the raw sludge. After treatment with the conditioner, the absorption peaks at 190–230 nm were significantly reduced, indicating that most of the aromatic compounds had been removed. The absorption peaks of the PAC and FA conditioned sludge filtrate at 200,230 nm exceed those of the original sludge, which may be due to the destruction of unsaturation during aromatics degradation [34]. The results show that fly ash coupled with PFS and fly ash coupled with PAC can effectively reduce organic acids and aromatic compounds in sludge filtrate.

### 3.8. Effect of Conditioner on Fractal Dimension of Sludge Floc

**Figure 8:**
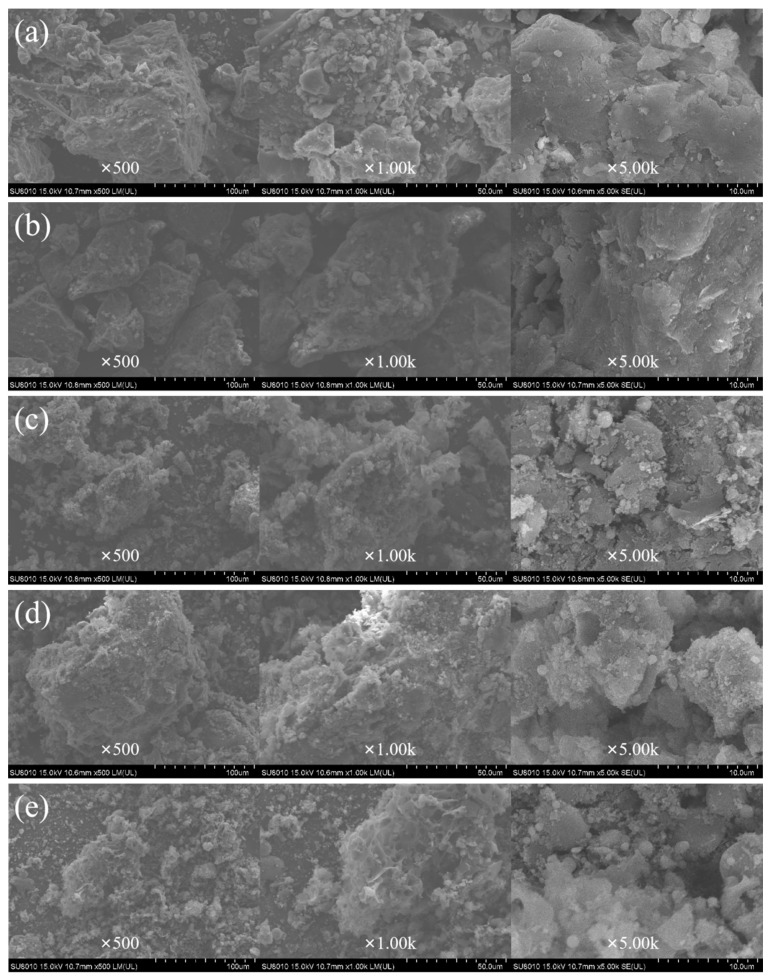
SEM figure of sludge conditioning with optimal dosage: (**a**) PFS, (**b**) PAC, (**c**) fly ash, (**d**) fly ash coupled with PFS, (**e**) fly ash coupled with PAC.

Figure 8 and Table 2 show the sludge cake with rough surface and tight internal structure after dehydration by PFS alone. The sludge cake prepared by fly ash alone has a loose structure and a spongy structure with spherical particles of different sizes on its surface. The sludge cake treated with PFS and PAC has a large number of pore structures and cracks and spherical particles on the surface. The sludge conditioned only by PAC and PFS has a dense structure, large floc volume, and less pore structure, which is not conducive to reducing the moisture content of the sludge cake. The surface of fly ash has many spherical particles with smooth surface. Microspheres play a crucial role in the adsorption of sludge flocs. The skeletal structure of fly ash maintains the permeability of the filter cake during compression and dehydration, which helps resist the deformation of the cake body, but the amount of fly ash is large. From the analysis of conditioning ability, to use only a single conditioner for sludge conditioning is not an ideal method of removing organic matter and sludge structure [35]. The pore structure of sludge conditioned by PAC-coupled fly ash and PFS-coupled fly ash was significantly improved and the number of pores increased, providing water filtration channels and reducing the compressibility of the sludge organic matter. PAC and PFS increase floc size through charge neutralization and net sweeping, which aids floc settling and drying [36]. Therefore, the fractal dimension of sludge conditioned by PAC-coupled fly ash and PFS-coupled fly ash is smaller. The results show that the sludge cake surface micromorphology results correspond to the sludge dewatering performance.

## 4. Conclusions

Fly ash can effectively reduce the moisture content of the sludge, and the fly ash dosage of 10 g/g DS can reduce the sludge specific resistance SRF to 1.03 × 10^11^ m/kg. After continuously increasing the fly ash dosage, it no longer had a significant impact on reducing the sludge specific resistance. The dosage of PAC and PFS, which can effectively reduce the sludge resistivity, is small when used as a single agent for sludge conditioning, but the moisture content of the filter cake is high. Considering the economic factors of sludge treatment and disposal, fly ash coupled with PAC and fly ash coupled with PFS were selected as composite conditioners for deep dewatering of sludge. The fly ash of 10 g/g DS coupled with PAC of 200 mg/g DS can reduce the sludge-specific resistance SRF, the moisture content of the filter cake, and the COD of the filtrate to 6.91 × 10^10^ m/kg, 76.10%, and 26 mg/L, respectively. The fly ash of 10 g/g DS coupled with the PFC of 200 mg/g DS can reduce the sludge-specific resistance SRF, the filter-cake moisture content, and the filtrate COD to 6.63 × 10^10^ m/kg, 76.39%, and 37 mg/L, respectively. Compared to a single conditioner, the addition of a compound conditioner can greatly improve the dewatering effect of the sludge and greatly reduce the amount of conditioner added, effectively increase the particle size of the floc, and reduce the organic matter in the filtrate. The effect on the pH of the sludge is small, which is beneficial for the subsequent treatment and disposal of the sludge and filtrate. Compared with the separate flocculation technology and oxidation technology, the skeleton-assisted flocculation method has the advantages of smaller chemical dosage and low cost. An improved dewatering system for activated sludge by scaffolding and flocculants was designed to provide a guide to practical engineering.

## Figures and Tables

**Table 1:** Original sludge quality index.

Sludge Index	Moisture Content of Raw Slduge (%)	Organic Matter Content (%)	SRF (m/kg)	pH Value
Numerical value	98.90	46.30	2.97 × 10^12^	7.51

**Table 2:** Fractal dimension of sludge cake under optimal dosage.

Conditioner	PFS	PAC	Fly Ash	Fly Ash +PFS	Fly Ash + PAC
Fractal dimension	2.03	1.99	1.96	1.91	1.86

## Data Availability

Data is contained within the article.
